# Supplementary material for: Identification and outcomes of acute kidney disease in patients presenting in Bolivia, Brazil, South Africa, and Nepal
Source: PLoS Med. 2024 Nov 14;21(11):e1004495. doi: 10.1371/journal.pmed.1004495 (PMC11611263; doi:10.1371/journal.pmed.1004495)
Supplement: S1 Text — Table A. Study sites. Table B. Clinical variables included in the logistic regression analysis used to create the risk score. Table C. Enrollment and demographic data at each study site. Table D. Presence of individual components of the AKD risk score in all patients and at each site. Table E. Multivariable analysis of factors associated with the development of AKD. Table F. Type of infection and main site of infection in patients with AKD in whom infection was a contributor to AKD development. Table G. Multivariable analysis of factors associated with mortality. Table H. Patient and kidney outcomes at healthcare facility discharge and at 90-day follow-up in patients with AKD with AKI. Table I. Prevalence, causes of kidney disease, and clinical outcomes in this and other studies within the 0by25 initiative. Fig A. Histograms of enrollment creatinine and eGFR in the entire cohort. (DOCX) [file pmed.1004495.s002.docx]

**Table A**: Study sites

|  | Boliva | Brazil | Nepal | South Africa |
| --- | --- | --- | --- | --- |
| Healthcare Centre (HCC) | CNS Comprehensive Family Medicine Center Quillacollo;  CNS Comprehensive Health Center Punata;  CNS Comprehensive Health Center Sacaba | Arapixuna HCC;  Tiningu HCC;  Alter do Chão HCC | Nil | Ezimpondweni clinic;  Hluhluwe clinic;  Mabibi clinic;  Manaba clinic;  Mbazwana clinic;  Mnqobokazi clinic;  Oqondweni clinic. All KwaZulu-Natal. |
| District Hospital | Hospital Central De Ivirgarzama;  Hospital Dr. Aurelio Melan Totora;  Hospital Municipal Autónomo de Colcapirhua | Hospital Municipal de Santarem | Nil | Mseleni Hospital, KwaZulu-Natal* |
| Tertiary Hospital | Hospital Obrero No 2, Cochabamba | Nil | B,P.Koirala Institute of Health Sciences, Dharan | Nil |

*not used as recruitment site, but follow-up undertaken here

**Table B**: Clinical variables included in the logistic regression analysis used to create the risk score

Features of Dehydration:

01.01 Diarrhea

01.02 Vomiting

01.03 Sweating

01.04 Increased thirst

01.05 Decreased intake

Features of Weakness:

13.01 Cannot walk

13.02 Cannot stand up

13.03 Localized in lower body

13.04 Localized in uppere body

13.05 Generalized

Urinary Symptoms:

02.01 Oliguria (low urine output)

02.02 Polyuria (too much urine)

02.03 Dysuria (painful urination)

02.04 Blood in urine

02.05 Finding of odor of urine

02.06 Incontinence

02.07 Urolith passed (stone)

Suspected Infection/Fever:

03.01 Fever [>= 38c (100.4F) or <= 36c (96.8F)]

03.02 Infective pharyngitis/tonsilitis

03.03 Upper respiratory infection

03.04 Infection of skin

03.05 Infectious disease of digestive tract

03.06 Malaria

03.07 Other Infection

Hypotension:

04.01 Low blood pressure

04.02 Shock (use of vasopressors)

04.03 Hemorrhage

Pain:

05.01 Head/Neck

05.02 Thorax

05.03 Abdomen

05.04 Lower limb

05.05 Upper limb

05.06 Joints/articulations

05.07 Pelvis

05.08 Whole body

05.09 Other

Swelling:

06.01 Entire body (anasarca)

06.02 Face/neck

06.03 Upper lime (arm)

06.04 Lower limb (leg)

06.05 Joins/articulations

06.06 Genital edema

06.07 Other

Traumatic Injury:

07.01 Entire body as a whole

07.02 Head/neck

07.03 Chest/thorax

07.04 Abdominen

07.05 Upper limb (arm)

07.06 Back

07.07 Lower limb (leg)

Allergic Reaction:

08.01 Drugs

08.02 Food

08.03 Pollen

08.04 Mold

08.05 Other

Poisoning:

09.01 Prescription drugs

09.02 Other drugs

09.03 Over the counter health food/herbal products

09.04 Pesticides

09.05 Other materials, e.g. hair dye, antifreeze (ethylele glycol)

Animal/Insect Bites:

10.01 Snake

10.02 Scorpion

10.03 Dog/cat

10.04 Bee/wasp

10.05 Spider

10.06 Other

Pregnancy and Delivery Related:

11.01 Suspected abortion

11.02 Vaginal discharge

11.03 Bleeding from vagina

11.04 Seizures

11.05 Coma

11.06 Headache

11.07 High blood pressure

11.08 Other

Additional Symptoms:

12.01 Asthenia

12.02 Dyspnea

12.03 Weight loss

12.04 Decreased appetite

12.05 Jaundice

12.06 Pallor

12.07 Coma

12.08 Acute confusion

12.09 Severe malnutrition

12.10 Other

**Table C:** Enrollment and demographic data at each study site

|  | Bolivia | Brazil | Nepal | South Africa | **All patients** |
| --- | --- | --- | --- | --- | --- |
| **Screening and enrollment** |  |  |  |  |  |
| Screened (n) | 1010 | 199 | 1963 | 1222 | **4394** |
| Excluded (n; % of screened) | 59 (5.8) | 2 (1.0) | 11 (0.6) | 11 (0.9) | **83 (1.9)** |
| Enrolled (n; % of screened) | 951 (94.2) | 197 (99.0) | 1952 (99.4) | 1211 (99.1) | **4311 (98.1)** |
| Enrolled with risk score ≥10 points (n; % of enrolled) | 951 (100.0) | 160 (81.2) | 1947 (99.7) | 132 (10.9) | **3190 (74.0)** |
| Enrolled on basis clinical judgment (risk score < 10 points) (n; % of enrolled) | 0 (0.0) | 37 (18.8) | 5 (0.3) | 1079 (89.1) | **1121 (26.0)** |
| Risk score (median; IQR) | 16 (13-20) | 15 (10-20) | 18 (14-22) | 2 (0-8) | **14 (8-19)** |
| Healthcare facility type where patient enrolled |  |  |  |  |  |
| Healthcare center | 113 (11.9) | 50 (25.4) | 0 (0.0) | 1193 (100.0) | **1356 (31.6)** |
| District Hospital | 531 (55.8) | 145 (73.6) | 0 (0.0) | 0 (0.0) | **676 (15.7)** |
| Tertiary Hospital | 307 (32.3) | 0 (0.0) | 1952 (100.0) | 0 (0.0) | **2259 (52.6)** |
| Other/missing | 0 (0.0) | 2 (1.0) | 0 (0.0) | 0 (0.0) | **2 (0.0)** |
| **Demographics** |  |  |  |  |  |
| Female (n; %) | 517 (54.4) | 88 (44.7) | 880 (45.1) | 804 (66.4) | **2289 (53.1)** |
| Age (median; IQR) | 56 (39-69) | 57 (42-69) | 60 (42-71) | 55 (43-66) | **57 (42-70)** |

**Table D**: Presence of individual components of the AKD risk score in all patients and at each site

|  | Bolivia n | Bolivia % | Brazil n | Brazil % | Nepal n | Nepal % | South Africa n | South Africa % | **All patients n** | **All patients %** |
| --- | --- | --- | --- | --- | --- | --- | --- | --- | --- | --- |
| 8 points appetite | 846 | 93.6 | 132 | 67.3 | 1887 | 96.9 | 373 | 32.8 | **3238** | **77.4** |
| 2 points weakness | 771 | 88.2 | 144 | 73.5 | 1880 | 96.6 | 239 | 21.0 | **3034** | **73.1** |
| 8 points oliguria | 336 | 43.0 | 71 | 36.8 | 306 | 16.0 | 66 | 5.8 | **779** | **19.3** |
| 4 points vomiting | 379 | 50.7 | 48 | 24.9 | 938 | 48.8 | 42 | 3.7 | **1407** | **35.2** |
| 2 points low oral fluid | 501 | 68.1 | 42 | 21.8 | 184 | 9.6 | 52 | 4.6 | **779** | **19.6** |
| 8 points hypotension | 179 | 26.4 | 34 | 17.5 | 704 | 36.7 | 38 | 3.4 | **955** | **24.3** |
| 5 points swelling | 249 | 32.3 | 104 | 53.9 | 672 | 35.1 | 66 | 5.9 | **1091** | **27.3** |

**Table E**: Multivariable analysis of factors associated with the development of AKD

| **Variable** | **Odds ratio** | **95% confidence interval** | **p-value** |
| --- | --- | --- | --- |
| Sex (male)* | 0.9172 | 0.7843 to 1.072 | 0.2782 |
| Age | 1.037 | 1.032 to 1.041 | <0.0001 |
| Total risk score | 1.028 | 1.013 to 1.043 | 0.0002 |
| Country (Bolivia)** | 0.3826 | 0.3123 to 0.4685 | <0.0001 |
| Country (Brazil)** | 0.3443 | 0.2433 to 0.4916 | <0.0001 |
| Country (South Africa)** | 0.08862 | 0.06732 to 0.1162 | <0.0001 |

*Reference for sex = female

**Reference for country = Nepal

**Table F**: Type of infection and main site of infection in patients with AKD in whom infection was a contributor to AKD development

**Type of infection**

|  | Bolivia n | Bolivia % | Brazil n | Brazil % | Nepal n | Nepal % | South Africa n | South Africa % | **Total n** | **Total %** |
| --- | --- | --- | --- | --- | --- | --- | --- | --- | --- | --- |
| Number with data | 420 |  | 64 |  | 757 |  | 35 |  | **1276** |  |
| Gastroenteritis | 132 | 31.4 | 5 | 7.8 | 217 | 28.7 | 11 | 31.4 | **365** | **28.6** |
| Malaria | 0 | 0.0 | 2 | 3.1 | 6 | 0.8 | 0 | 0.0 | **8** | **0.6** |
| Leptospirosis | 0 | 0.0 | 0 | 0.0 | 4 | 0.5 | 0 | 0.0 | **4** | **0.3** |
| TB | 22 | 5.2 | 0 | 0.0 | 3 | 0.4 | 0 | 0.0 | **25** | **2.0** |
| Dengue | 6 | 1.4 | 0 | 0.0 | 27 | 3.6 | 0 | 0.0 | **33** | **2.6** |
| Other bacterial | 226 | 53.8 | 56 | 87.5 | 499 | 65.9 | 24 | 68.6 | **805** | **63.1** |
| Other viral | 34 | 8.1 | 1 | 1.6 | 1 | 0.1 | 0 | 0.0 | **36** | **2.8** |

**Site of infection**

|  | Bolivia n | Bolivia % | Brazil n | Brazil % | Nepal n | Nepal % | South Africa n | South Africa % | **Total n** | **Total %** |
| --- | --- | --- | --- | --- | --- | --- | --- | --- | --- | --- |
| Number with data | 418 |  | 64 |  | 728 |  | 38 |  | **1248** |  |
| Pneumonia | 93 | 22.2 | 4 | 6.3 | 142 | 19.5 | 1 | 2.6 | **240** | **19.2** |
| Meningitis | 2 | 0.5 | 1 | 1.6 | 7 | 1.0 | 0 | 0.0 | **10** | **0.8** |
| Urinary Tract | 152 | 36.4 | 11 | 17.2 | 336 | 46.2 | 24 | 63.2 | **523** | **41.9** |
| GI Tract | 144 | 34.4 | 21 | 32.8 | 223 | 30.6 | 13 | 34.2 | **401** | **32.1** |
| Septicaemia | 15 | 3.6 | 1 | 1.6 | 4 | 0.5 | 0 | 0.0 | **20** | **1.6** |
| Soft Tissue | 7 | 1.7 | 26 | 40.6 | 14 | 1.9 | 0 | 0.0 | **47** | **3.8** |
| Skin | 5 | 1.2 | 0 | 0.0 | 2 | 0.3 | 0 | 0.0 | **7** | **0.6** |

**Table G**: Multivariable analysis of factors associated with mortality

1. In-hospital death

| **Variable** | **Odds ratio** | **95% confidence interval** | **p-value** |
| --- | --- | --- | --- |
| Sex (male)* | 0.7895 | 0.5009 to 1.235 | 0.0002 |
| Age | 1.025 | 1.012 to 1.039 | <0.0001 |
| Total risk score | 1.136 | 1.100 to 1.173 | <0.0001 |
| Country (Bolivia)** | 29.83 | 13.95 to 77.47 | 0.0002 |
| Country (Brazil)** | 10.30 | 2.901 to 35.01 | <0.0001 |
| Country (South Africa)** | 14.62 | 4.443 to 49.41 | 0.0123 |
| Presence of AKD | 2.478 | 1.269 to 5.327 | 0.0002 |

1. Death at any time up to 90-day follow-up

| **Variable** | **Odds ratio** | **95% confidence interval** | **p-value** |
| --- | --- | --- | --- |
| Sex (male)* | 1.046 | 0.8270 to 1.325 | 0.7073 |
| Age | 1.025 | 1.018 to 1.032 | <0.0001 |
| Total risk score | 1.072 | 1.050 to 1.094 | <0.0001 |
| Country (Bolivia)** | 0.6752 | 0.5078 to 0.8890 | 0.0059 |
| Country (Brazil)** | 0.6399 | 0.3462 to 1.095 | 0.1258 |
| Country (South Africa)** | 0.1258 | 0.05083 to 0.2672 | <0.0001 |
| Presence of AKD | 1.215 | 0.8544 to 1.767 | 0.2912 |

*Reference for sex = female

**Reference for country = Nepal

**Table H**: Patient and kidney outcomes at healthcare facility discharge and at 90-day follow-up in patients with AKD with AKI

|  | Bolivia | Brazil | Nepal | South Africa | **Total** |
| --- | --- | --- | --- | --- | --- |
| **At healthcare facility discharge** | | | | | |
| Number with data | 116 | 14 | 404 | 100 | **634** |
| Unknown (no creatinine after enrolment) | 15 (12.9) | 2 (14.3) | 262 (64.9) | 53 (53.0) | **332 (52.4)** |
| Partial recovery | 44 (37.9) | 6 (42.9) | 34 (8.4) | 3 (3.0) | **87 (13.7)** |
| Complete recovery | 42 (36.2) | 3 (21.4) | 104 (25.7) | 41 (41.0) | **190 (30.0)** |
| No Kidney recovery | 8 (6.9) | 1 (7.1) | 3 (0.7) | 2 (2.0) | **14 (2.2)** |
| Died | 7 (6.0) | 2 (14.3) | 1 (0.2) | 1 (1.0) | **11 (1.7)** |
| **At 90-day follow-up** | | | | | |
| Number with follow-up | 94 | 9 | 334 | 13 | **450** |
| Death after discharge | 0 (0.0) | 1 (11.1) | 47 (14.1) | 0 (0.0) | **48 (10.7)** |
| Death during admission or post discharge follow-up | 7 (7.4) | 3 (33.3) | 48 (14.4) | 1 (7.7) | **7 (13.1)** |
| Number with creatinine at follow-up | 94 | 5 | 286 | 13 | **398** |
| Partial recovery | 36 (38.3) | 4 (80.0) | 96 (33.6) | 5 (38.5) | **141 (35.4)** |
| Complete recovery | 57 (60.6) | 0 (0.0) | 171 (59.8) | 7 (53.8) | **235 (59.0)** |
| No Kidney recovery | 1 (1.1) | 1 (20.0) | 19 (6.6) | 1 (7.7) | **22 (5.5)** |

**Table I:** Prevalence, causes of kidney disease, and clinical outcomes in this and other studies within the 0by25 initiative

|  | ***Current Study*** | ***0by25 Feasibility Study (Macedo et al, PLOS Medicine, 2020)*** | ***0by25 Global Snapshot (Mehta et al, Lancet, 2016)*** |
| --- | --- | --- | --- |
| **Study overview** | Observational cohort study, 2018-2020.  Healthcare centres and hospitals in Bolivia, Brazil, Nepal, and South Africa. | Observational cohort study, | Cross-sectional study, Sept –Dec 2014; snapshot undertaken on 3 index days  289 centres in 72 countries |
| **Patient population** | Adult patients at risk of kidney disease as determined by a risk score and clinical judgment.  N=4311 | Adult and paediatric patients at risk of kidney disease as determined by a risk score  N=2101 | Adult and paediatric patients with confirmed AKI. Screening undertaken from all patients under clinician care at that time.  N=4018 |
| **Prevalence of kidney disease** | Kidney disease: 68.6%  AKD with and without AKI: 67.8%  *kidney disease determined at hospital discharge; urinalysis data not captured | Kidney disease: 69%  AKD with and without AKI: 66%  *kidney disease determined at 7 days; urinalysis data captured | N/A |
| **Causes of kidney disease** | Infection 44.0%  Hypotension/shock 19.3%  Dehydration 19.1% | N/A | In LLMICs:  Sepsis 39%; Infection 36%  Dehydration 46%  Hypotension/shock 38% |
| **Kidney follow-up and outcomes** | At 90-day follow up (n=1865 AKD patients):  Persistent kidney disease 48.4%; reclassification as CKD 39.7% | At 90-day follow up (n=275 AKD patients):  Reclassification as CKD 50.5% | AKI outcome at last in-hospital observation:  Complete recovery 28%  Partial recovery 36% |
| **Patient follow-up and outcomes** | At discharge (n=2922 AKD patients):  Mortality: 2.9%  At 90-day follow-up (n= 2119 AKD patients):  AKD patient mortality (after discharge): 9.7% | At discharge (n=869 hospitalised AKD patients)  Mortality: 9.0%  At 3-month follow up (n=1310 AKD patients)  AKD patient mortality (after discharge): 10.3% | AKI Mortality at 7 days:  12% LLMICs and 10% HICs |

AKD – acute kidney disease; AKI – acute kidney injury; N/A – not available; CKD – chronic kidney disease; LLMIC – low- and low-middle income country; HIC – high income country.

**Fig A**: Histograms of enrollment creatinine and eGFR in the entire cohort
