# Supplementary material for: Identification and outcomes of acute kidney disease in patients presenting in Bolivia, Brazil, South Africa, and Nepal
Source: PLoS Med. 2024 Nov 14;21(11):e1004495. doi: 10.1371/journal.pmed.1004495 (PMC11611263; doi:10.1371/journal.pmed.1004495)
Supplement: Attachment — Submitted filename: Reply to reviewers.docx [file pmed.1004495.s003.docx]

We thank the editorial team and the reviewers for considering our paper, and for their helpful suggestions and support. Responses to comments are outlined below. Line numbers for changes have been given, and these refer to the line numbers in the tracked manuscript.

Comments from the academic editor:

1. Interpretation of the data is limited by a problem of selection bias at every stage of the study. 98% of those screened were deemed to be at high risk for AKD, implying a high degree of selection. Follow-up after discharge occurred in only 59% and repeat creatinine testing occurred in 64% of those with AKD. In addition it is clear that there was a big difference in practice in the centre in South Africa versus the other centres. The impact of this problem of selection bias should be discussed in more detail in the limitations section.

Thank you for highlighting this. We agree and we have updated the limitations section as you helpfully suggest highlighting the issue of differences between sites, and of selection bias (lines 492-502). We have also highlighted the impact of the enrolled cohort on the interpretation of the performance of the risk score, as highlighted in point 4. The following has been added to the limitations. “We designed this pragmatic observational study to assess AKD identification and outcomes as part of routine healthcare. This was undertaken in a variety of healthcare facilities managed within different health systems across 3 continents. This led to marked heterogeneity in clinical practice and outcomes between sites (e.g. significantly less AKD in South Africa; lower inpatient mortality in Nepal) and results should be interpreted with this in mind. Further, selection bias likely occurred during cohort identification, given the high proportion (98%) of patients deemed to be at AKD risk, and there may have been selection bias in those patients with AKD that were followed up. The performance of the risk score reported is based on this pre-selected cohort, and this may not be representative of its performance in a broader population. ..”

2. The paper would benefit from more discussion of the concept of AKD and why it is particularly relevant in low resource settings.

Thank you for this helpful suggestion. Further background data has been added to the introduction to highlight the importance of AKD and its management (see response to reviewer 1, point 2 – lines 72-85). We have also included a discussion around the concept of AKD as it differs according to the KDIGO and ADQI definitions (see response to reviewer 2, general comment and points 3 and 4 – lines 469-477).

3. The study is reported to have recruited persons “presenting” to healthcare centres but it is implied that all were admitted. The authors should clarify this and perhaps specify that all participants were admitted.

Thank you for seeking clarification on this. Not all patients recruited were admitted to the healthcare facility for an inpatient stay. All patients underwent serum creatinine testing on presentation, some were admitted, and some were discharged after treatment on the same day. We do not have data on facility length of stay and have added this as a limitation (line 504). We also refer to outcomes ‘at the end of healthcare facility admission’. To clarify this, we have added the following to the methods (lines 192-196): ‘Of note, in the manuscript we refer to healthcare ‘admission’ which includes both patients who attended a healthcare facility, underwent SCr testing (+- the relevant management) and were discharged on the same day, in addition to those patients that were admitted for an inpatient stay.’

4. The ROC curve in this case was based only on data from a highly selected group of patients considered to be at high risk. Performance in this population would not necessarily be representative of performance in a broader population.

Thank you for highlighting this. It has been added to the limitations – please see response to point 1, above.

5. More detail should be presented on the multivariable analyses.

More details have been given. Please see response to review 1 (point 7 – lines 246-254), and reviewer 3 (point 6).

-----------------------------------------------------------
Comments from the reviewers:

Reviewer #1: The study evaluates the performance of a risk score for Acute Kidney Disease (AKD) identification and outcomes in low- and low-middle-income countries (LLMICs). Conducted across healthcare facilities in Bolivia, Brazil, South Africa, and Nepal from September 2018 to November 2020, adult patients were screened using a symptom-based risk score and clinical judgment. Serum creatinine testing confirmed AKD in 2922 out of 4311 at-risk patients (67.8%). The risk score's area under the curve was 0.73, indicating moderate accuracy. AKD prevalence was higher when using the risk score compared to clinical judgment alone (80.2% vs. 32.5%). Mortality during admission was 2.9% for AKD patients, and 9.7% after discharge, with 48.4% of AKD patients showing persistent kidney disease at follow-up. The study underscores the risk score's utility in AKD identification and the need for diligent follow-up in LLMICs.
Although the study was carefully designed and a lot of data were collected over the study period, I found the methods section, particularly the statistical methods section, to be lacking. Below are my specific comments.

1. Please add line numbers for easier reference.

Line numbers have been added

2. Introduction: The introduction mentions that kidney disease disproportionately affects disadvantaged populations in LLMICs but lacks specific statistics or data to quantify this burden. The authors could include concrete data or statistics on the prevalence and impact of kidney disease in LLMICs to strengthen the argument and provide a clearer picture of the problem's scale.

Thank you for this helpful suggestion. Further details have been added to the introduction (lines 72-85).

3. Introduction: The pilot study results are mentioned, but the challenges faced, such as the high rate of loss to follow-up, are not discussed in depth. Consider providing a more detailed analysis of the pilot study's challenges, including reasons for the high loss to follow-up rate and potential strategies to mitigate this issue in future implementations.

Thank you for highlighting this. We have expanded on this in the relevant section of the introduction (lines 109-116)

4. Introduction: The introduction briefly mentions the establishment of the KCN but lacks details on its implementation and scope. It could be helpful to include more information about the KCN's implementation, such as the specific interventions, the roles of participating healthcare facilities, and how the updated AKD risk score is integrated into routine clinical care.

Thank you for your interest in the implementation of the KCN approach. Brief details of the implementation of the protocol for AKD management are given in the methods section (study design, settings, and participants). We feel a full description of its implementation is beyond the scope of this article, which is focused on the use of the risk score in addition to patient outcomes post AKD. We plan to publish a separate article that outlines implementation of the project in more detail.

5. Method, Study Design, Setting, and Participants: It would be beneficial to provide a detailed description of the regression used to predict the risk score. Specifically, what type of regression was used and outline the variables included in the regression models.

The risk score was developed using logistic regression. Variables included in this model are listed below. This analysis was done using data from the 0by25 feasibility project. These details have been added to the methods section (lines 167-170), and a further supplementary table (Supplementary 2) added listing the variables included in the model.

Features of Dehydration:

01.01 Diarrhea

01.02 Vomiting

01.03 Sweating

01.04 Increased thirst

01.05 Decreased intake

Features of Weakness:

13.01 Cannot walk

13.02 Cannot stand up

13.03 Localized in lower body

13.04 Localized in uppere body

13.05 Generalized

Urinary Symptoms:

02.01 Oliguria (low urine output)

02.02 Polyuria (too much urine)

02.03 Dysuria (painful urination)

02.04 Blood in urine

02.05 Finding of odor of urine

02.06 Incontinence

02.07 Urolith passed (stone)

Suspected Infection/Fever:

03.01 Fever [>= 38c (100.4F) or <= 36c (96.8F)]

03.02 Infective pharyngitis/tonsilitis

03.03 Upper respiratory infection

03.04 Infection of skin

03.05 Infectious disease of digestive tract

03.06 Malaria

03.07 Other Infection

Hypotension:

04.01 Low blood pressure

04.02 Shock (use of vasopressors)

04.03 Hemorrhage

Pain:

05.01 Head/Neck

05.02 Thorax

05.03 Abdomen

05.04 Lower limb

05.05 Upper limb

05.06 Joints/articulations

05.07 Pelvis

05.08 Whole body

05.09 Other

Swelling:

06.01 Entire body (anasarca)

06.02 Face/neck

06.03 Upper lime (arm)

06.04 Lower limb (leg)

06.05 Joins/articulations

06.06 Genital edema

06.07 Other

Traumatic Injury:

07.01 Entire body as a whole

07.02 Head/neck

07.03 Chest/thorax

07.04 Abdominen

07.05 Upper limb (arm)

07.06 Back

07.07 Lower limb (leg)

Allergic Reaction:

08.01 Drugs

08.02 Food

08.03 Pollen

08.04 Mold

08.05 Other

Poisoning:

09.01 Prescription drugs

09.02 Other drugs

09.03 Over the counter health food/herbal products

09.04 Pesticides

09.05 Other materials, e.g. hair dye, antifreeze (ethylele glycol)

Animal/Insect Bites:

10.01 Snake

10.02 Scorpion

10.03 Dog/cat

10.04 Bee/wasp

10.05 Spider

10.06 Other

Pregnancy and Delivery Related:

11.01 Suspected abortion

11.02 Vaginal discharge

11.03 Bleeding from vagina

11.04 Seizures

11.05 Coma

11.06 Headache

11.07 High blood pressure

11.08 Other

Additional Symptoms:

12.01 Asthenia

12.02 Dyspnea

12.03 Weight loss

12.04 Decreased appetite

12.05 Jaundice

12.06 Pallor

12.07 Coma

12.08 Acute confusion

12.09 Severe malnutrition

12.10 Other

6. Outcome Measures: It would be helpful to categorize the outcomes into primary and secondary outcomes. Additionally, specify the clinical variables and outcomes compared between the two groups. A detailed description of these variables and outcomes would enhance clarity.

Thank you for this suggestion. Primary and secondary outcomes have been outlined in the relevant section of the methods; clinical variables and outcomes have been clarified (lines 225-235).

7. Statistical Methods: Provide more details about the exact multivariable analysis performed to determine the factors associated with AKD and mortality. Clarify if logistic regression was used and explain why certain variables (e.g., age, sex, country of enrollment) were included in the models. Describe the selection process for these variables. Additionally, explain Youden's index in the main text for better understanding. Overall, the statistical methods section needs more comprehensive information.

Thank you for asking for this clarification. Logistic regression was used. The clinical variables were selected as these data were pre-specified as required in all participants at study enrollment, and due to differences in these variables in patients with AKD and in patient survival in univariable analyses. These details have been added to the statistical methods section (lines 246-254) in addition to an explanation of Youden’s index (lines 257-259).

Reviewer #2: In this study, the authors attempted to validate the performance of the AKD risk stratification tool. According to reference 10, the initial design and rationale of this AKD tool are based on the pre-hospital kidney disease classification (NKD, AKD, CKD). This AKD concept might slightly differ from the AKD concept used in large cohort studies or from the concept according to the ADQI consensus (non-recovery status after AKI). However, considering prior exposure to a community-acquired AKI episode, this concept might still be applicable.

Thank you for highlighting the ADQI concept of AKD being kidney disease that persists after an episode of AKI, subsequently defined as CKD if kidney disease is persistent for >90 days. This was highlighted in a consensus statement in 2017. We agree with this concept of the continuum of acute and chronic kidney diseases, and the somewhat arbitrary cut-offs in terms of duration of disease required for each definition. We refer to this continuum within the current article. To confirm, we use an updated concept of AKD as outlined by KDIGO in a consensus statement from 2021 (*Lameire, N. H. et al. Harmonizing acute and chronic kidney disease definition and classification: report of a Kidney Disease: Improving Global Outcomes (KDIGO) Consensus Conference. Kidney Int. 100, 516–526 (2021*). This simplifies the concept of AKD, defined by ‘abnormalities of kidney function and/or structure with a duration of < 3 months’. This includes the subset of patients that fulfil the KDIGO definition of AKI. If kidney disease persists beyond 3 months, this then becomes CKD.

Comment 1: Should Table 2 be cited as Table 1 (with the current Table 1 changing to Table 2) according to the main text in the manuscript?

Thank you for spotting this typo. It has been corrected.

Comment 2: According to reference 18, the authors decided to use baseline creatinine based on "The latest SCr documented prior to healthcare facility admission and the lowest SCr during healthcare facility admission were used to determine the baseline SCr; an imputed baseline SCr based on an assumed eGFR was not used." Did the authors consider using the lowest creatinine level from -7 to -365 days before this index admission as baseline creatinine, as well?

Thank you for highlighting the issue of determining baseline creatinine values. As highlighted in the following reference, there is marked heterogeneity in how this is done across studies of AKI (Siew, E. D. & Matheny, M. E. Choice of Reference Serum Creatinine in Defining Acute Kidney Injury. *Nephron* **131**, 107–112 (2015). Moreover, as highlighted in our paper, a baseline creatinine is unavailable in most patients managed in LLMICs, a consequence largely of a lack of electronic health records, and a lack of routine health screening. The reference above highlights the potential value of a lowest, or perhaps even better, a mean creatinine in days 7-365 prior to admission as the baseline value. We do not feel this is practical in the routine care of patients in LLMICs, as firstly a baseline value if often unavailable (multiple values are very rare) and secondly the timing of any value is often unclear. Given this project collected data from patients managed as part of routine clinical care, we undertook the pragmatic approach of using the latest creatinine prior to hospital admission without time restriction as one of the criteria to determine the baseline creatinine value.

Comment 3: I understand the authors followed the KDIGO AKD concept. However, I suggest that the authors also discuss the slightly different concept of AKD from ADQI. As mentioned above, the score used to identify patients at risk for AKD might account for an under-diagnosed community-acquired AKI episode prior. The authors should also cite the ADQI reference in this article.
ref 1: Acute kidney disease and renal recovery: consensus report of the Acute Disease Quality Initiative (ADQI) 16 Workgroup. Nat Rev Nephrol. 2017 Apr;13(4):241-257. doi: 10.1038/nrneph.2017.2

Thank you for this reference. A discussion around the KDIGO and ADQI concepts of AKD is outlined above. The discussion section has been updated to include this and the ADQI reference cited (lines 469-477). The definitions section has also been modified for clarity (lines 213-214).

Comment 4: In the discussion section, I suggest the authors also discuss the post-AKD follow-up and the differences in patient outcomes in this study compared to other prior studies. Additionally, please cite some recently published articles provided below.
ref 1: Unveiling the enigma of acute kidney disease: predicting prognosis, exploring interventions, and embracing a multidisciplinary approach. Kidney Res Clin Pract. 2024 Jul;43(4):406-416. doi: 10.23876/j.krcp.23.289
ref 2: Incidence and Transition of Acute Kidney Injury, Acute Kidney Disease to Chronic Kidney Disease after Acute Type A Aortic Dissection Surgery. J Clin Med. 2021 Oct 18;10(20):4769.
ref 3: Comprehensive versus standard care in post-severe acute kidney injury survivors, a randomized controlled trial. Crit Care. 2021 Aug 31;25(1):322. doi: 10.1186/s13054-021-03747-7
ref 4: Acute Kidney Disease After Acute Decompensated Heart Failure. Kidney Int Rep. 2022 Jan 3;7(3):526-536. doi: 10.1016/j.ekir.2021.12.033
ref 4: Quality of Care for Acute Kidney Disease: Current Knowledge Gaps and Future Directions. Kidney Int Rep. 2020 Aug 6;5(10):1634-1642. doi: 10.1016/j.ekir.2020.07.031

Thank you for these suggestions. The references have been cited (lines 474 and 477). We have expanded our discussion on the potential role of post-AKD clinics, specifically around the lack of knowledge / current strategies that improve outcomes post AKD episode (lines 477-480). The outcomes in this study are compared to previous largely studies of AKD in LLMICs in table 7.

Comment 5: For the causes of AKD listed in Table 5, could the authors provide supplemental table or appendix information regarding how each etiology for AKD, such as infection, cardiorenal, and hepatorenal, was defined?

The contributors and causes of AKD were determined by the clinical judgement of the treating clinician. This clarification has been added to table 5.

Comment 6: Could the authors also provide the long-term kidney outcomes (such as the need for maintenance dialysis) or MAKE-90 after identifying AKD patients in this cohort? (since the authors mentioned "We undertook follow-up at 90 days")

Thank you for highlighting the importance of longer-term outcomes in this cohort. 12 patients required ongoing maintenance dialysis at 90 days. This represents 0.6% of AKD patients who were followed-up to this timepoint. This detail has been added / clarified within the results section (lines 361-362).

Comment 7: The 90-day mortality after AKD is reported as "13.7%." This seems a little high. Do the authors have further discussion or explanation, with more detailed information? (since the in-hospital rate only 2.9% ?)

Thank you for highlighting this. This was indeed a somewhat unexpected finding in our study, albeit very similar post discharge mortality rates were seen in the feasibility study (10.3%). The reasons for the high post discharge mortality rate are unclear – cause of death was not recorded and is a limitation of the current study. However, the finding warrants further investigation, and this consistent finding across more than one study, adds further support for the need to follow AKD patients closely after the initial episode. This has been added within the discussion (lines 454-456).

Comment 8: According to reference 10, the AKI episode after admission might be considered a recurrent AKI episode or AKD/AKI progression following a previously unidentified community AKI episode (as shown in reference 10, Fig 1: the AKI develops and is identified after AKD). Did the authors use the same concept in this article (AKD first, then AKI)? If so, please clarify this more clearly in the introduction and methods section since, as mentioned above, this differs from the ADQI AKD concept.

Thank you. As highlighted above the KDIGO AKD concept (used by us) differs from the ADQI concept. As clarified in the definitions section (and table 2), the KDIGO concept of AKD is broad incorporating any abnormality of kidney function present with a duration of <3 months. Within this broad AKD definition will be a subset of patients that fulfil the definition of AKI (AKD with AKI), and other patients that have AKD without AKI. In contrast to the Macedo et al study, we report kidney disease classification at a single timepoint (end of healthcare facility admission) as opposed to the 2-stage approach (enrolment and then 7 days) employed in the previous study.

Reviewer #3: This is a very interesting and important work performed by Evans et al. The researchers included adult patients who were at increased risk of AKD and who had a creatinine measurement performed.

Thank you for your support

Few queries

1)      Could you please clarify why the multivariable analysis includes the presence of AKD in the model if the study aims to compare clinical variables and outcomes between those with AKD and those without kidney disease (NKD)?

Thank you for asking for clarification on the variables included. The presence of AKD was only included in the model for mortality. Age, sex, country of enrolment, and risk score were included in models for both AKD development and mortality. This has been clarified in the text (lines 248-251), in addition to an explanation of the choice of these variables, as outlined to reviewer 1.

2)      If the inclusion criteria was availability of creatinine measurement, I am unsure why 28.7% of patients had creatinine prior to enrolment - "Those at increased kidney disease risk who underwent SCr testing were included. Patients on dialysis or with a kidney transplant, and those with missing data for presenting SCr or age category, were excluded."

Apologies for any confusion here. The serum creatinine required for enrolement was that undertaken at the time when the patient was enrolled. The creatinine prior to enrolment refers to previous historical creatinine measurements undertaken prior to the current healthcare episode. Only 28.7% of patients had a historical creatinine available. As discussed in the responses above, the relatively lack of historical creatinine’s (which may act as baseline measurements for the diagnosis of AKI) is common in LLMICs, largely due to a lack of routine health screening and electronic health records. We have clarified the text the relevant section of the results (line 284).

3)      Can Table 3 show number of patients please.

Patient numbers have been added.

4)      I am unclear with regards to serum creatinine measurement. Did the author measure serum creatinine by POC at enrolment for patients who did not have pre-enrolment creatinine? Or, some patients had creatinine measure twice - pre-enrolment and at the time of enrolment?

Thank you for asking for clarity over the creatinine measurements at enrolment. As outlined in response to point 2, all patients had a creatinine measured at study enrolement. In 73% of patients this was done by POC device. Of all patients enrolled, 28.7% had an historical serum creatinine available.

5)      Authors need to provide a brief on Education and training program delivered to healthcare workers.

Thank you for your interest in the education and training program. The training was site specific and included face-face workshops delivered over multiple days. These were run by local nephrologists, and attended by multidisciplinary healthcare professionals (clinicians, nurses, physician assistants) providing clinical care at the sites of project implementation. These details have been added to the methods section (lines 160-164). A full description of the KCN approach is beyond the scope of this paper, and will be reported within a separate article.

6)      The multivariable analysis has been included as supplementary table instead of being in the main document.

Thank you for highlighting this. We shared a concern about the number of tables, as mentioned below, and hence included these tables in the supplementary. We have added odds ratios and 95% confidence intervals for each significant variable within the main text (lines 294-2297, 330-334, and 352-355).

Additional minor comment:
1)      There are too many tables

As outlined in the response to point 6 above, we have tried to balance having important results within the main manuscript against over burdening the reader with too much data / too many tables. We feel the balance is reasonable in the updated manuscript.
