# Supplementary material for: Identification and outcomes of acute kidney disease in patients presenting in Bolivia, Brazil, South Africa, and Nepal
Source: PLoS Med. 2024 Nov 14;21(11):e1004495. doi: 10.1371/journal.pmed.1004495 (PMC11611263; doi:10.1371/journal.pmed.1004495)
Supplement: Attachment — Submitted filename: Reply (second) to reviewers.docx [file pmed.1004495.s004.docx]

We thank the reviewers, and editorial team, for their further review.

------------------------------------------------------------
ACADEMIC EDITOR COMMENTS

The authors have responded comprehensively and adequately to the reviewers' comments. The study still has multiple limitations but importantly these are now more completely acknowledged and discussed. There are a few typo's to correct and I picked up two minor language issues as follows:

Line76-77:  “…high mortality from the acute AKD episode…” suggest rephrase to “…high mortality from the acute episode…” or “…high mortality from the AKD episode…”

Line 93-94: “This challenge is a consequence, at least in part, due to a deficiency in nephrology education…” suggest rephrase to: “This challenge is a consequence, at least in part, of a deficiency in nephrology education…”

Thank you. The above have been altered.
------------------------------------------------------------
Requests from Editors:

Please note that we always require a point-by-point response to not only reviewer comments, but all editorial comments, including general editorial requests. Please be sure to provide such a document.

FINANCIAL DISCLOSURE

The funding statement should include: specific grant numbers, initials of authors who received each award, URLs to sponsors’ websites. Also, please state whether any sponsors or funders (other than the named authors) played any role in study design, data collection and analysis, the decision to publish, or preparation of the manuscript. If they had no role in the research, include this sentence: “The funders had no role in study design, data collection and analysis, decision to publish, or preparation of the manuscript.”

Section updated.

DATA AVAILABILITY

The Data Availability Statement (DAS) requires revision. For each data source used in your study:

a) If the data are freely or publicly available, note this and state the location of the data: within the paper, in Supporting Information files, or in a public repository (include the DOI or accession number).

b) If the data are owned by a third party but freely available upon request, please note this and state the owner of the data set and contact information for data requests (web or email address). Note that a study author cannot be the contact person for the data.

c) If the data are not freely available, please describe briefly the ethical, legal, or contractual restriction that prevents you from sharing it. Please also include an appropriate contact (web or email address) for inquiries (again, this cannot be a study author).

Section updated

CODE AVAILABILITY

We expect all researchers with submissions to PLOS in which author-generated code underpins the findings in the manuscript to make all author-generated code available without restrictions upon publication of the work. In cases where code is central to the manuscript, we may require the code to be made available as a condition of publication. Authors are responsible for ensuring that the code is reusable and well documented. Please make any custom code available, either as part of your data deposition or as a supplementary file. Please add a sentence to your data availability statement regarding any code used in the study, e.g. "The code used in the analysis is available from Github [URL] and archived in Zenodo [DOI link]" Please review our guidelines at <https://gbr01.safelinks.protection.outlook.com/?url=https%3A%2F%2Fjournals.plos.org%2Fplosmedicine%2Fs%2Fmaterials-software-and-code-sharing&data=05%7C02%7Crhys.evans5%40nhs.net%7C4561b625aa3248dac0ae08dce2d3f684%7C37c354b285b047f5b22207b48d774ee3%7C0%7C0%7C638634647227849638%7CUnknown%7CTWFpbGZsb3d8eyJWIjoiMC4wLjAwMDAiLCJQIjoiV2luMzIiLCJBTiI6Ik1haWwiLCJXVCI6Mn0%3D%7C0%7C%7C%7C&sdata=UhkBvrq5SkNux99D1rNfmLXJeury9hsu4VeweI6Q814%3D&reserved=0> and ensure that your code is shared in a way that follows best practice and facilitates reproducibility and reuse. Because Github depositions can be
readily changed or deleted, we encourage you to make a permanent DOI'd copy (e.g. in Zenodo) and provide the URL.

Not applicable

TITLE

We suggest changing the title to “Identification and outcomes of acute kidney disease in patients presenting in Bolivia, Brazil, South Africa and Nepal”.

Changed

ABSTRACT

1) Abstract: Please structure your abstract using the PLOS Medicine headings (Background, Methods and Findings, Conclusions). Please combine the Methods and Findings sections into one section.

Changed

2) l. 36: Please define ‘AKD’ at first use.

Done

3) In the last sentence of the Abstract Methods and Findings section, please describe the main limitation(s) of the study's methodology.

Added

AUTHOR SUMMARY

We ask that you include a short, non-technical Author Summary of your research to make findings accessible to a wide audience that includes both scientists and non-scientists. The Author Summary should immediately follow the Abstract in your revised manuscript. This text is subject to editorial change and should be distinct from the scientific abstract. Ideally each sub-heading should contain 2-3 single sentence, concise bullet points containing the most salient points from your study. In the final bullet point of 'What Do These Findings Mean?', please include the main limitations of the study in non-technical language. Please see our author guidelines for more information: <https://gbr01.safelinks.protection.outlook.com/?url=https%3A%2F%2Fjournals.plos.org%2Fplosmedicine%2Fs%2Frevising-your-manuscript%23loc-author-summary&data=05%7C02%7Crhys.evans5%40nhs.net>%7C4561b625aa3248dac0ae08dce2d3f684%7C37c354b285b047f5b22207b48d774ee3%7C0%7C0%7C638634647227869162%7CUnknown%7CTWFpbGZsb3d8eyJWIjoiMC4wLjAwMDAiLCJQIjoiV2luMzIiLCJBTiI6Ik1haWwiLCJXVCI6Mn0%3D%7C0%7C%7C%7C&sdata=oME0iAhPf5kUkXvG%2FBwhmvBTUDKswhR6MQvfQ%2F%2FUIow%3D&reserved=0.

This has been added

INTRODUCTION

1) Please cite the reference numbers in square brackets. Citations should precede punctuation. Please revise throughout the entire manuscript.

The references have been formatted according to the Vancouver style (with square as opposed to curved brackets) – as per guidance in the link given below.

2) Please remove any subheadings from the Introduction section.

Done

METHODS AND RESULTS

1) Please state in the Methods section whether the study had a prospective protocol or analysis plan. If a prospective analysis plan (from your funding proposal, IRB or other ethics committee submission, study protocol, or other planning document written before analyzing the data) was used in designing the study, please include the relevant document(s) with your revised manuscript as a Supporting Information file to be published alongside your study and cite it in the Methods section. A legend for this file should be included at the end of your manuscript. If no such document exists, please make sure that the Methods section transparently describes when analyses were planned, and when/why any data-driven changes to analyses took place. Changes in the analysis, including those made in response to peer review comments, should be identified as such in the Methods section of the paper, with rationale.

A formal prospective analysis plan was not used. Analyses were determined after data collection. This has been added to the methods (statistics section).

2) Please ensure that the study is reported according to the STROBE guideline (please use RECORD if you feel it is more appropriate), and include the completed STROBE checklist as Supporting Information. Please add the following statement, or similar, to the Methods: "This study is reported as per the Strengthening the Reporting of Observational Studies in Epidemiology (STROBE) guideline (S1 Checklist)."
When completing the checklist, please use section and paragraph numbers, rather than page numbers.

Sentence has been added and STROBE checklist uploaded.

3) l.247: We suggest introducing the abbreviation ‘CI’ for ‘confidence intervals’ here.

Done

4) l.265, please state the statistical meaning of the numbers in parentheses, e.g. "57 (interquartile range (IQR): 42-70) years". Please revise throughout.

Done (and abbreviation for IQR added to statistics section when first used)

5) l.290ff: Throughout, we suggest reporting statistical information as follows to improve clarity for the reader "22% (95% CI [13%,28%]; p</=)". When reporting 95% CIs please separate upper and lower bounds with commas instead of hyphens as the latter can be confused with reporting of negative values. For example: “(OR 1.04, 95% CI [1.03-1.04])”

Done

6) Table 1: Please spell out ‘AKD’ in the title or define ‘AKD’ below the table.

Done

7) Table 3: Please define ‘POC’ and ‘IQR’ below the table.

Done

8) Table 4: Please spell out ‘AKD’ and ‘NKD’ in the title or define both below the table. Please add a unit for age (years). Please define ‘HCC’, ‘IQR’. Please change the first demographic to “Sex, Female (n; %)”.

Done

9) Table 5: Please spell out ‘AKD’ in the title or define ‘AKD’ below the table. Please define ‘HIV’ and ‘IV’. Please provide a definition of the numerical values (e.g. n (%)).

Done

10) Table 6: Please spell out ‘AKD’ in the title or define ‘AKD’ below the table. Please provide a definition of the numerical values (e.g. n (%)).

Done

11) Table 7: Please define ‘AKD’, ‘AKI’, ‘N/A’, ‘CKD’, ‘LLMIC’ and ‘HIC’.

Done

12) Please provide titles and legends for all figures (including those in Supporting Information files).

These have been added after the competing interests section.

13) Figure 1: Please define ‘AKD’, ‘KRT’, ‘NKD’, ‘CKD’.

Done

14) Figure 2: Please define ‘AKD’ and ‘NKD’. Please indicate in the figure caption the meaning of the bars and whiskers as well as the lines and dots. Please add a brief explanation on the risk score.

Done

DISCUSSION

1) Please remove any subheadings from the Discussion section.

Done

2) l.396: Please temper claims of primacy of results by stating, "to our knowledge" or something similar.

Thank you. This has been altered.

3) Please note that we prefer not to introduce new tables/figures in the Discussion section. We suggest moving Table 7 to the Supplementary Material.

We have moved table to supplementary.

REFERENCES

1) PLOS uses the numbered citation (citation-sequence) method and first six authors, et al.

2) Please ensure that journal name abbreviations match those found in the National Center for Biotechnology Information (NCBI) databases (<https://gbr01.safelinks.protection.outlook.com/?url=http%3A%2F%2Fwww.ncbi.nlm.nih.gov%2Fnlmcatalog%2Fjournals&data=05%7C02%7Crhys.evans5%40nhs.net%7C4561b625aa3248dac0ae08dce2d3f684%7C37c354b285b047f5b22207b48d774ee3%7C0%7C0%7C638634647227886092%7CUnknown%7CTWFpbGZsb3d8eyJWIjoiMC4wLjAwMDAiLCJQIjoiV2luMzIiLCJBTiI6Ik1haWwiLCJXVCI6Mn0%3D%7C0%7C%7C%7C&sdata=gSHsum0SiNXzkcBc8YUffEjC49XqOly5W1cBdsAMyHE%3D&reserved=0>), and are appropriately formatted and capitalised.

3) Where website addresses are cited, please include the complete URL and specify the date of access (e.g. [accessed: 12/06/2024]).

4) Please also see <https://gbr01.safelinks.protection.outlook.com/?url=https%3A%2F%2Fjournals.plos.org%2Fplosmedicine%2Fs%2Fsubmission-guidelines%23loc-references&data=05%7C02%7Crhys.evans5%40nhs.net%7C4561b625aa3248dac0ae08dce2d3f684%7C37c354b285b047f5b22207b48d774ee3%7C0%7C0%7C638634647227904001%7CUnknown%7CTWFpbGZsb3d8eyJWIjoiMC4wLjAwMDAiLCJQIjoiV2luMzIiLCJBTiI6Ik1haWwiLCJXVCI6Mn0%3D%7C0%7C%7C%7C&sdata=11SexHvRy%2FT%2F18bbKRvW8t3z5JZs8a%2FSiB8SSbfSzWQ%3D&reserved=0> for further details on reference formatting.

References have been reformatted in the ‘Vancouver style’ as the guidance at point 4 above.

SUPPLEMENTARY MATERIAL

1) Please note that supplementary material will be posted as supplied by the authors. Therefore, please amend it according to the relevant comments outlined here and in the previous decision letter.

The supplementary file has been updated as suggested.

2) In the published article, supporting information files are accessed only through a hyperlink attached to the captions. For this reason, you must list captions at the end of your manuscript file. You may include a caption within the supporting information file itself, as long as that caption is also provided in the manuscript file. Do not submit a separate caption file.

When SI files are contained with a single file:
Please label the file as ‘S1 Supporting Information’.
Please apply alphabetical labelling to each table and figure contained within the S1 file. For example, ‘Fig A’ to ‘Fig Z’ and ‘Table A’ to ‘Table Z’.
Plain text does not need to be labelled and can just be given a title as necessary. For example, ‘Statistical Analysis Plan’.
Please cite tables/figures as ‘Fig A in S1 Supporting Information’ and/or ‘Table A in S1 Supporting Information’, for example.
Please cite plain text as, ‘Statistical Analysis Plan in S1 Supporting Information’, for example.

The file has been renamed and Tables / Figures relabelled as above.

When SI files are uploaded as separate files:
Please label tables as ‘S1 Table’ (so on) and figures as ‘S1 Fig’ (and so on).
Any additional documents (protocols/analysis plans etc.) can be labelled as ‘S1 Protocol’, for example. Please cite items as exactly as labelled.

SOCIAL MEDIA

To help us extend the reach of your research, please provide any X (formerly known as Twitter) handle(s) that would be appropriate to tag, including your own, your co-authors’, your institution, funder, or lab. Please enter in the submission form any handles you wish to be included when we post about this paper.

Please tag the following: @rhysdrevans, @RenalUCL, @RClaure_nefro, @ISNkidneycare

------------------------------------------------------------
Comments from Reviewers:

Reviewer #1: I would like to thank the authors for their thoughtful revisions in response to my and the other reviewers' comments. After reviewing the authors' responses and the revised manuscript, I am satisfied that they have adequately addressed the concerns raised. I am pleased to recommend this paper for acceptance and publication.

Thank you

Reviewer #2: The authors have addressed my previous question regarding the different AKD definitions compared to the ADQI consensus and the recent KDIGO criteria. Additionally, detailed information about MAKE-90 and the etiologies of AKD/AKI has been provided in the revised manuscript.

Thank you

Any attachments provided with reviews can be seen via the following link:


------------------------------------------------------------

General Editorial Requests

1) We ask every co-author listed on the manuscript to fill in a contributing author statement. If any of the co-authors have not filled in the statement, we will remind them to do so when the paper is revised. If all statements are not completed in a timely fashion this could hold up the re-review process. Should there be a problem getting one of your co-authors to fill in a statement we will be in contact. YOU MUST NOT ADD OR REMOVE AUTHORS UNLESS YOU HAVE ALERTED THE EDITOR HANDLING THE MANUSCRIPT TO THE CHANGE AND THEY SPECIFICALLY HAVE AGREED TO IT.

No author changes have been made

2) Please ensure that the paper adheres to the PLOS Data Availability Policy (see <https://gbr01.safelinks.protection.outlook.com/?url=http%3A%2F%2Fjournals.plos.org%2Fplosmedicine%2Fs%2Fdata-availability&data=05%7C02%7Crhys.evans5%40nhs.net%7C4561b625aa3248dac0ae08dce2d3f684%7C37c354b285b047f5b22207b48d774ee3%7C0%7C0%7C638634647227921939%7CUnknown%7CTWFpbGZsb3d8eyJWIjoiMC4wLjAwMDAiLCJQIjoiV2luMzIiLCJBTiI6Ik1haWwiLCJXVCI6Mn0%3D%7C0%7C%7C%7C&sdata=guWXuuZf6JKXvgEdmE0oMAO8czM1HTm%2FWOn2jZ5wweo%3D&reserved=0>), which requires that all data underlying the study's findings be provided in a repository or as Supporting Information. For data residing with a third party, authors are required to provide instructions with contact information for obtaining the data. PLOS journals do not allow statements supported by "data not shown" or "unpublished results." For such statements, authors must provide supporting data or cite public sources that include it.

Contact instructions have been provided

3) Please review your reference list to ensure that it is complete and correct. If you have cited papers that have been retracted, please include the rationale for doing so in the manuscript text, or remove these references and replace them with relevant current references. Any changes to the reference list should be mentioned in the rebuttal letter that accompanies your revised manuscript.

Reference list has been reviewed and no changes needed.
