# Supplementary material for: Identification and outcomes of acute kidney disease in patients presenting in Bolivia, Brazil, South Africa, and Nepal
Source: PLoS Med. 2024 Nov 14;21(11):e1004495. doi: 10.1371/journal.pmed.1004495 (PMC11611263; doi:10.1371/journal.pmed.1004495)
Supplement: Attachment — Submitted filename: Reply (third) to reviewers.docx [file pmed.1004495.s005.docx]

Thank you for asking for clarification on the below points.

1.      Abstract, ll 55-58: Please clarify the denominators used to calculate the percentages for persistent kidney disease (48.4%) and reclassification (39.7%). Should the denominator be the 2119 patients with AKD who had follow up (per line 368 in the Results)? If so, the percentage with persistent kidney disease would be 42.6% (902 of 2119), and the percentage who were reclassified would be 34.9% (740 of 2119). Please check and clarify if needed, and please accept my apologies if I have missed something.

The kidney outcomes are reported as percentages out of the number of patients that underwent kidney follow up. This was 1865 (see figure 1, also line 380). I have made a short addition to abstract to clarify this.

2.      Please format your Author summary using bullet points, rather than a continuous narrative. Each sub-heading should contain 2-3 single sentence, concise bullet points containing the most salient points from your study. In the final bullet point of ‘What Do These Findings Mean?’, please include the main limitations of the study in non-technical language. Please see our author guidelines for more information: <https://gbr01.safelinks.protection.outlook.com/?url=https%3A%2F%2Fjournals.plos.org%2Fplosmedicine%2Fs%2Frevising-your-manuscript%23loc-author-summary&data=05%7C02%7Crhys.evans5%40nhs.net>%7Cb749e6083a514f6e997408dcec507d26%7C37c354b285b047f5b22207b48d774ee3%7C0%7C0%7C638645077646960037%7CUnknown%7CTWFpbGZsb3d8eyJWIjoiMC4wLjAwMDAiLCJQIjoiV2luMzIiLCJBTiI6Ik1haWwiLCJXVCI6Mn0%3D%7C0%7C%7C%7C&sdata=X8lqYGXGxktmmYNGgZisVKYeif8e8dAXPIi2VYtJq0Q%3D&reserved=0.

This section has been reformatted.

3.      Results, ll 294-297; Please report the denominator used to calculate these percentages and explain why this is different from the total number (n=4311). Is this due to missing data for type of center visited?

Thanks for asking for clarification here. Data were missing in 18 patients. The denominator (n=4293) has been added to this section for clarity.

4.      Financial disclosure statement: please include grant numbers and the initials of the author to whom the grants were awarded.

Funding was provided to the ISN as an organisation. This was received by its executive director in 2017, Luca Segantini. Funds for the project were subsequently managed by KH on behalf of the ISN. There was no grant number associated with the project. I have uploaded a copy of the grant award for your information. I have also adjusted the financial disclosure section so above is clear.

5.      STROBE checklist – please remove page numbers (leaving only line numbers) as these will not correspond to the final published document

Page numbers have been removed, and line numbers updated to reflect updated manuscript.
